# Human Vascular Endothelial Cells Promote the Secretion of Vascularization Factors and Migration of Human Skin Fibroblasts under Co-Culture and Its Preliminary Application

**DOI:** 10.3390/ijms232213995

**Published:** 2022-11-13

**Authors:** Tian Hou, Miaomiao Du, Xiang Gao, Meiwen An

**Affiliations:** College of Biomedical Engineering, Taiyuan University of Technology, Taiyuan 030024, China

**Keywords:** cell co-culture, human skin fibroblasts, human vascular endothelial cells, tissue engineering scaffolds, cell migration, vascularization factor

## Abstract

The good treatment of skin defects has always been a challenge in the medical field, and the emergence of tissue engineering skin provides a new idea for the treatment of injured skin. However, due to the single seed cells, the tissue engineering skin has the problem of slow vascularization at the premonitory site after implantation into the human body. Cell co-culture technology can better simulate the survival and communication environment of cells in the human body. The study of multicellular co-culture hopes to bring a solution to the problem of tissue engineering. In this paper, human skin fibroblasts (HSFs) and human vascular endothelial cells (HVECs) were co-cultured in Transwell. The Cell Counting Kit 8 (CCK8), Transwell migration chamber, immunofluorescence, Western blot (WB), and real time quantitative PCR (RT-qPCR) were used to study the effects of HVECs on cell activity, migration factor (high mobility group protein 1, HMGB1) and vascularization factor (vascular endothelial growth factor A, VEGFA and fibroblast growth factor 2, FGF2) secretion of HSFs after co-cultured with HVECs in the Transwell. The biological behavior of HSFs co-cultured with HVECs was studied. The experimental results are as follows: (1) The results of cck8 showed that HVECS could promote the activity of HSFs. (2) HVECs could significantly promote the migration of HSFs and promote the secretion of HMGB1. (3) HVECs could promote the secretion of VEGFA and FGF2 of HSFs. (4) The HVECs and HSFs were inoculated on tissue engineering scaffolds at the ratio of 1:4 and were co-cultured and detected for 7 days. The results showed that from the third day, the number of HSFs was significantly higher than that of the control group without HVECs.

## 1. Introduction

The wound healing of skin has always been a basic problem in surgery [1], tissue-engineered skin provides a better way for the treatment of skin tissue defects [2]. With the clinical application of tissue-engineered skin, its disadvantages are gradually emerging. First of all, the vascularization of tissue-engineered skin takes a long time [3]. Second, the seed cells of tissue engineering generally select stem cells with high differentiation ability [4,5], the source of cells is relatively simple. Finally, a single cell cannot simulate the living environment of cells in the body, which makes it difficult for the function of skin tissue.

The formation of granulation tissue plays a key role in the process of wound healing. Granulation tissue is mainly composed of a large number of fibroblasts and capillaries. As the main cells of granulation tissue, fibroblasts proliferation and secretion of growth factors play a crucial role in wound healing [6,7]. Therefore, fibroblasts from a wide range of sources (the main cells of dermis) were selected as the seed cells of tissue engineering and studied in this paper [8]. At the same time, in order to solve the problem of slow vascularization of engineering in the group, it is necessary to look for cells related to angiogenesis. Vascular endothelial cells are the main constituent cells of capillaries and play the most important role in the process of vascularization, so it is very important to introduce vascular endothelial cells into tissue engineering scaffolds to study the vascularization of scaffolds. Cell co-culture technology is to co-culture two or more kinds of cells. Compared with single cell culture, cell co-culture can better simulate the living environment of cells in vivo [9].

Based on this, in this paper, HSFs were used as the research objects, and HVECs were used as co-culture cells to establish a Transwell non-contact co-culture model. The effect of HVECs on the biological behavior of HSFs was studied. The study mainly focused on the effects of HVECs on the migration and vascularization factors of HSFs in co-culture (the recognized vascularization factors are VEGFA and FGF2 [10]). To select the ratio of endothelial cells to fibroblasts suitable for tissue engineering co-culture, this condition was detected on three-dimensional chitosan demethylcellulose sodium scaffold to verify the growth of HSFs. The purpose of this study is to provide an experimental theoretical basis for the treatment of skin defects by tissue engineering.

## 2. Results

### 2.1. Effect of Cell Co-Culture on the Activity of HSFs

**Figure 1:**
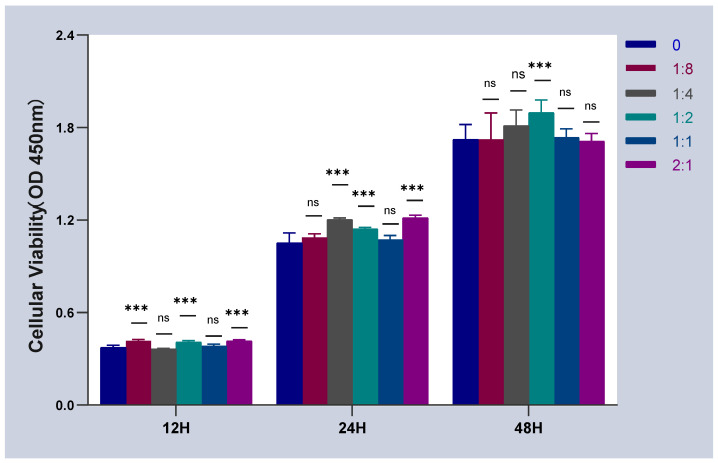
The cell activity curves of HSFs, when HSFs were cell co-cultured with HVECs. ns: *p* > 0.05, ***: *p* < 0.001.

The activity of co-cultured HSFs was detected by CCK-8. The result is shown in Figure 1. When HSFs were co-cultured with HVECs, the co-culture time was 12 h, and compared with the control group (HVCEs: HSFs = 0), the activity of HSFs was not significantly inhibited by HVECs. Moreover, HVCEs also promote the activity of HSFs to a certain extent. This conclusion is also applicable when the co-culture time is 24 h and 48 h.

### 2.2. Effect of Cell Co-Culture on the Migration Ability of HSFs

#### 2.2.1. Results of Transwell Cell Migration Experiment

**Figure 2:**
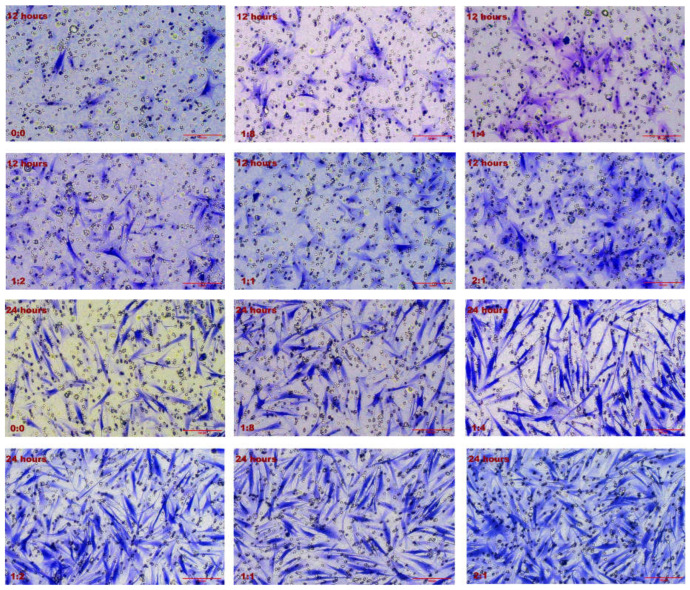

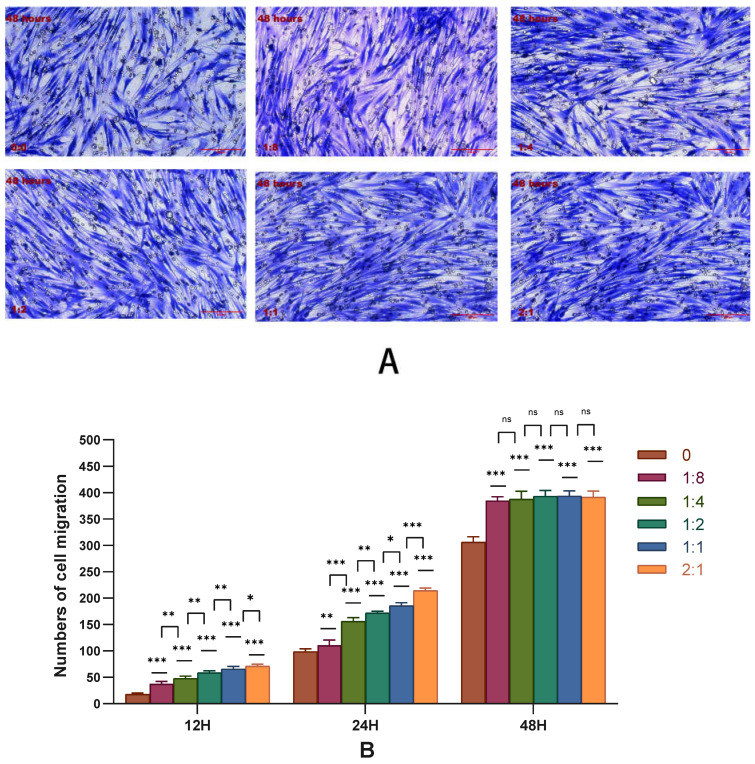
Migration of HSFs after 12 h, 24 h, and 48 h co-culture. (**A**) The crystal violet staining map of HSFs after migrating through Transwell chamber. (**B**) The statistical chart of fine migration of fibers. ns: *p* > 0.05, *: *p* < 0.05, **: *p* < 0.01, ***: *p* < 0.001. Scale bar: 100 μm.

Transwell was used to detect the migration ability of HSFs in the co-culture system, and the results are shown in Figure 2. Figure 2A shows the Transwell cell migration diagram of HSFs. After 12 h of cell co-culture, it can be obviously observed that the migration number of HSFs increases with the increase of the number of HVECs. The number of HSFs migration increased with the extension of co-culture time. The number of HSFs that migrated through the Transwell chamber was counted, and the results are shown in Figure 2B. According to the data analysis, when the cell co-culture time was 12 h and 24 h, the migration number of HSFs increased significantly with the increase of the proportion of HVECs. In each culture period (the culture time is 12 h and 24 h respectively), there are significant differences between the experimental group (HVECs:HSFs = 1:8, HVECs:HSFs = 1:4, HVECs:HSFs = 1:2, HVECs:HSFs = 1:1, HVECs:HSFs = 2:1) and the control group (HVECs:HSFs = 0), and there are also significant differences between the two adjacent experimental groups (HVECs:HSFs = 1:8, HVECs:HSFs = 1:4, HVECs:HSFs = 1:2, HVECs:HSFs = 1:1, HVECs:HSFs = 2:1). When the cell co-culture time was 48 h, there was a significant difference in the number of HSFs migration between experimental groups (HVECs:HSFs = 1:8, HVECs:HSFs = 1:4, HVECs:HSFs = 1:2, HVECs:HSFs = 1:1, HVECs:HSFs = 2:1) and the control group (HVECs:HSFs = 0). However, there was no significant difference between the two adjacent experimental groups (HVECs:HSFs = 1:8, HVECs:HSFs = 1:4, HVECs:HSFs = 1:2, HVECs:HSFs = 1:1, HVECs:HSFs = 2:1). Through the Transwell migration experiment, it can be found that as long as there are HVECs in the cell co-culture system, compared with the single HSFs culture, the migration of HSFs will be significantly promoted.

#### 2.2.2. Migration Factor-HMGB1 Was Detected by RT-qPCR

**Figure 3:**
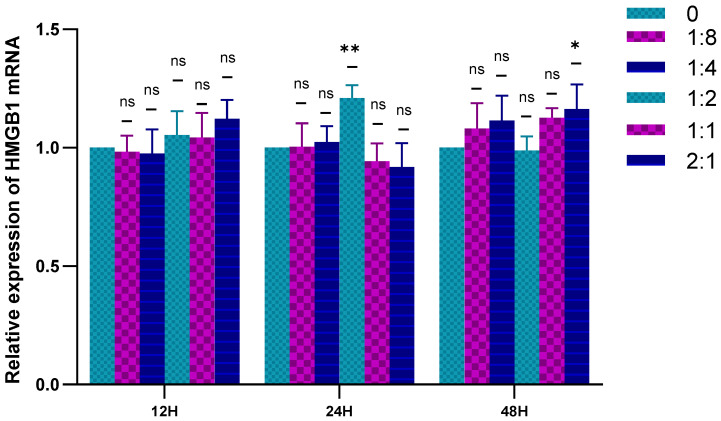
Statistical diagram of relative expression of HMGB1 in HSFs in cell co-culture. ns: *p* > 0.05, *: *p* < 0.05, **: *p* < 0.01.

The RT-qPCR was used to detect the expression of HSFs HMGB1 in the cell co-culture system, and the results are shown in Figure 3. When the cells are co-cultured for 12 h, HVECs had no significant effect on the expression of HMGB1 in HSFs. When the cell co-culture time was 24 h and the co-culture ratio was 1:2, HVECs could significantly promote the expression of HMGB1 in HSFs. When the cell co-culture time was 48 h and the cell co-culture ratio was 2:1, HVECs could also significantly promote the expression of HMGB1 in HSFs. In general, HVECs promote the expression of HMGB1 in HSFs.

#### 2.2.3. Detection of HSFs Migration Factor HMGB1 by Immunofluorescence

**Figure 4:**
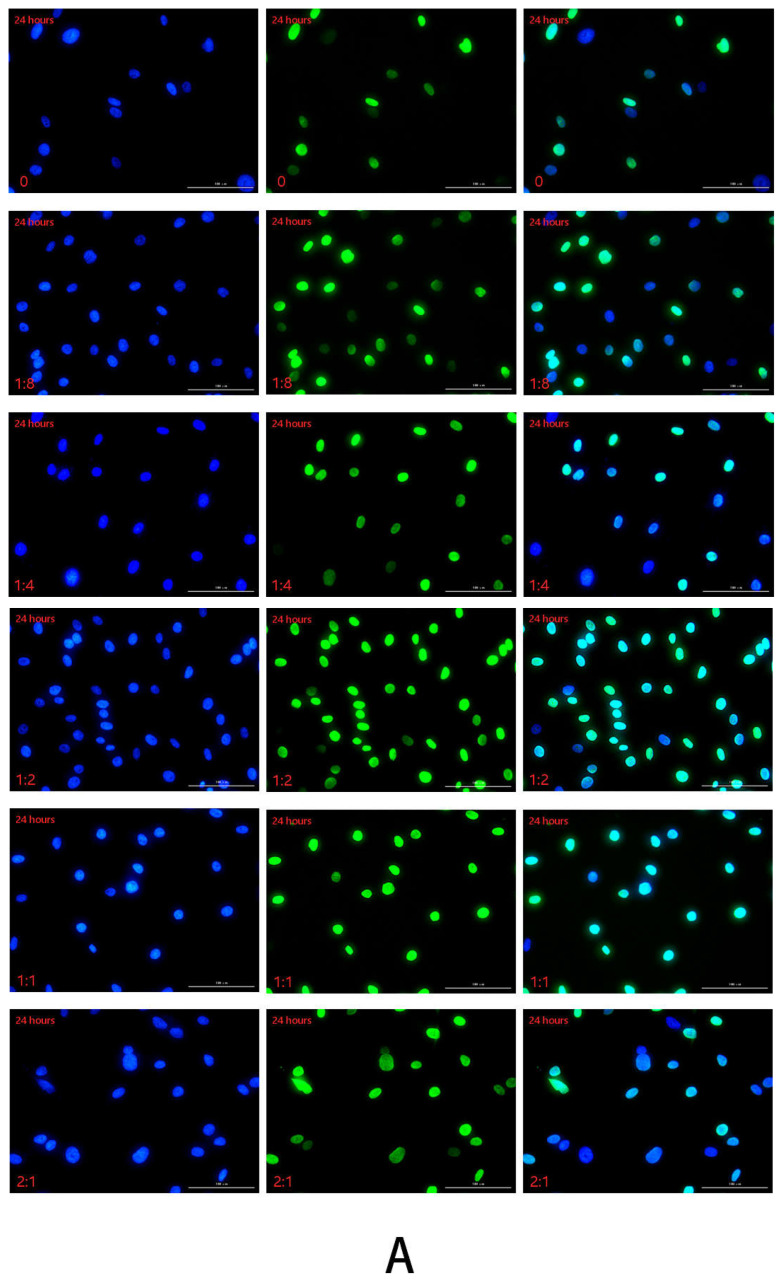

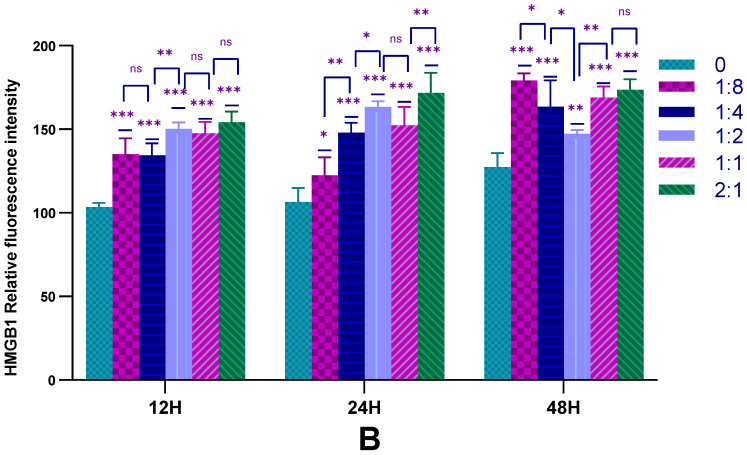
In the cell co-culture system, HSFs were stained with immunofluorescence. (**A**) The immunofluorescence staining of HMGB1 protein secreted by HSFs after cell co-culture for 24 h. Scale bar: 100 μm. (**B**) The fluorescence intensity of HMGB1 protein secreted by HSFs after cell co-culture for 12 h, 24 h, and 48 h. ns: *p* > 0.05, *: *p* < 0.05, **: *p* < 0.01, ***: *p* < 0.001.

The immunofluorescence technique was used to detect the HMGB1 protein secreted by HSFs in the cell co-culture system, and the results are shown in Figure 4, Figures S1 and S2. In this experiment, the cell co-culture time was 12 h, 24 h, 48 h, respectively. In each culture period, the control group was HVECs:HSFs = 0, and the experimental group was HVECs:HSFs = 1:8, HVECs:HSFs = 1:4, HVECs:HSFs = 1:2, HVECs:HSFs = 1:1, HVECs:HSFs = 2:1. The green fluorescence part is the HMGB1 protein secreted by HSFs stained by immunofluorescence (Figure 4A), and the blue is the HSFs nucleus stained by DAPI (Figure 4A). As can be seen from the Figure 4A, the HMGB1 protein secreted by HSFs is only concentrated in the nucleus, and there is no HMGB1 protein in the cytoplasm. The fluorescence intensity statistics of the green fluorescence parts in Figure 4A, Figures S1 and S2 were carried out, and the results are shown in Figure 4B. According to the statistical diagram of fluorescence intensity, when the cell co-culture time was 12 h, with the increase of the proportion of HVECs, the secretion of HMGB1 protein of HSFs showed a significant upward trend. The cell co-culture time was 24 h and 48 h, and its phenomenon was the same at 12 h. The results of immunofluorescence showed that the secretion of HMGB1 protein in HSFs increased significantly with the increase in the number of HVECs.

#### 2.2.4. Detection of HSFs Migration Factor HMGB1 by WB

**Figure 5:**
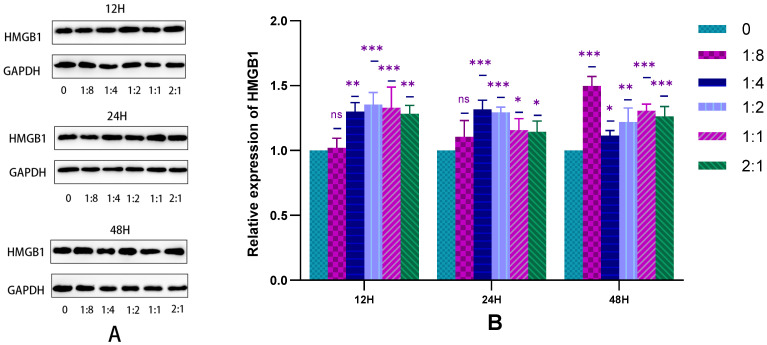
In the cell co-culture system, the WB detection map of HMGB1 protein secreted by HSFs. (**A**) The WB map of HMGB1 protein secreted by HSFs after cell co-culture for 12 h, 24 h, and 48 h. (**B**) The statistical results of relative expression of HMGB1 protein secreted by HSFs. ns: *p* > 0.05, *: *p* < 0.05, **: *p* < 0.01, ***: *p* < 0.001.

The WB was used to detect the HMGB1 protein secreted by HSFs in the cell co-culture system, and the results are shown in Figure 5. From the statistical results of Figure 5B, the HVECs can significantly promote the secretion of HMGB1 protein in HSFs under different co-culture time. This is consistent with the results of the Transwell migration experiment, indicating that there is a positive relationship between the increase of HMGB1 protein and the migration of HSFs. The results showed that HVECs could significantly promote the secretion of HMGB1 by HSFs when it was co-cultured with HSFs.

### 2.3. Detection of HSFs Vascularization Factor-VEGFA and FGF2

#### 2.3.1. The Expression of HSFs VEGFA and FGF2 Detected by RT-qPCR

**Figure 6:**
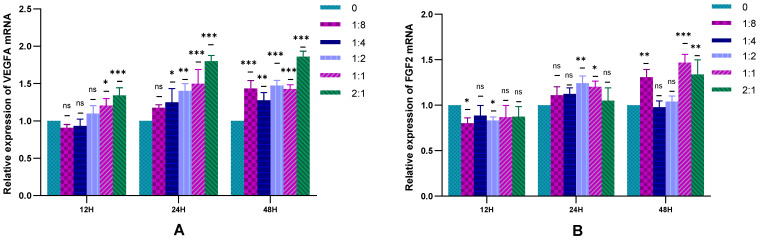
In the cell co-culture system, the RT-qPCR statistical diagram of HSFs VEGFA and FGF2 expression. (**A**) The relative expression of VEGFA mRNA in HSFs. (**B**) The relative expression of FGF2 mRNA in HSFs. ns: *p* > 0.05, *: *p* < 0.05, **: *p* < 0.01, ***: *p* < 0.001.

The RT-qPCR technique was used to detect the gene expression of VEGFA and FGF2 secreted by HSFs in the cell co-culture system. The results are shown in Figure 6. It can be seen from Figure 6A that the presence of HVECs in the cell co-culture system can promote the expression of the VEGFA gene in HSFs, which was more obvious when HSFs were cell co-cultured with a high proportion of HVECs for 24 h and 48 h. It can be seen from Figure 6B that there was almost no significant effect of HVECs on the expression of FGF2 in HSFs when the cell co-culture time was 12 h. When the cell co-culture time increased, HVECs could promote the expression of HSFs FGF2 to some extent. The results showed that when the co-culture time of HSFs and HVECs was prolonged, for example, from 12 h to 24 h and 48 h, HVECs could significantly promote the secretion of VEGFA and FGF2 of HSFs. Therefore, in order to achieve the role of HVECs in promoting the secretion of vascularization factors by HSFs, the cell co-culture time of 24 h and 48 h should be selected in cell co-culture.

#### 2.3.2. The Secretion of HSFs Proteins VEGFA and FGF2 Detected by Immunofluorescence

**Figure 7:**
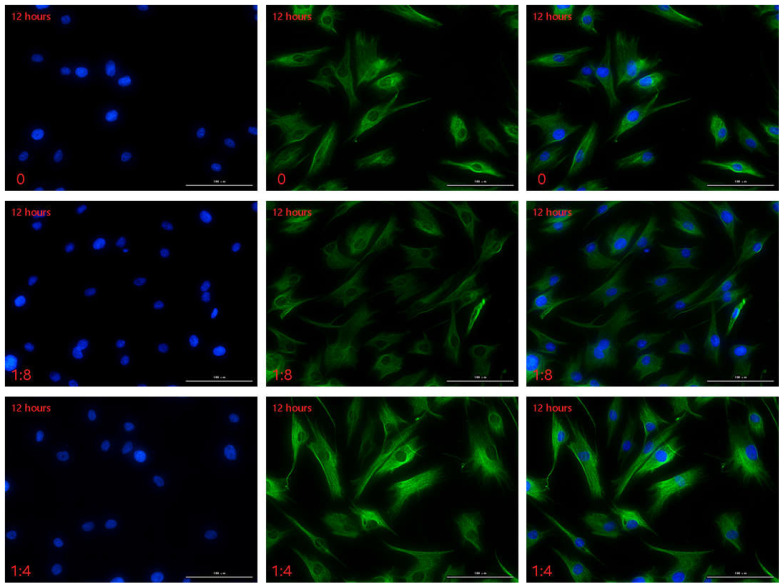

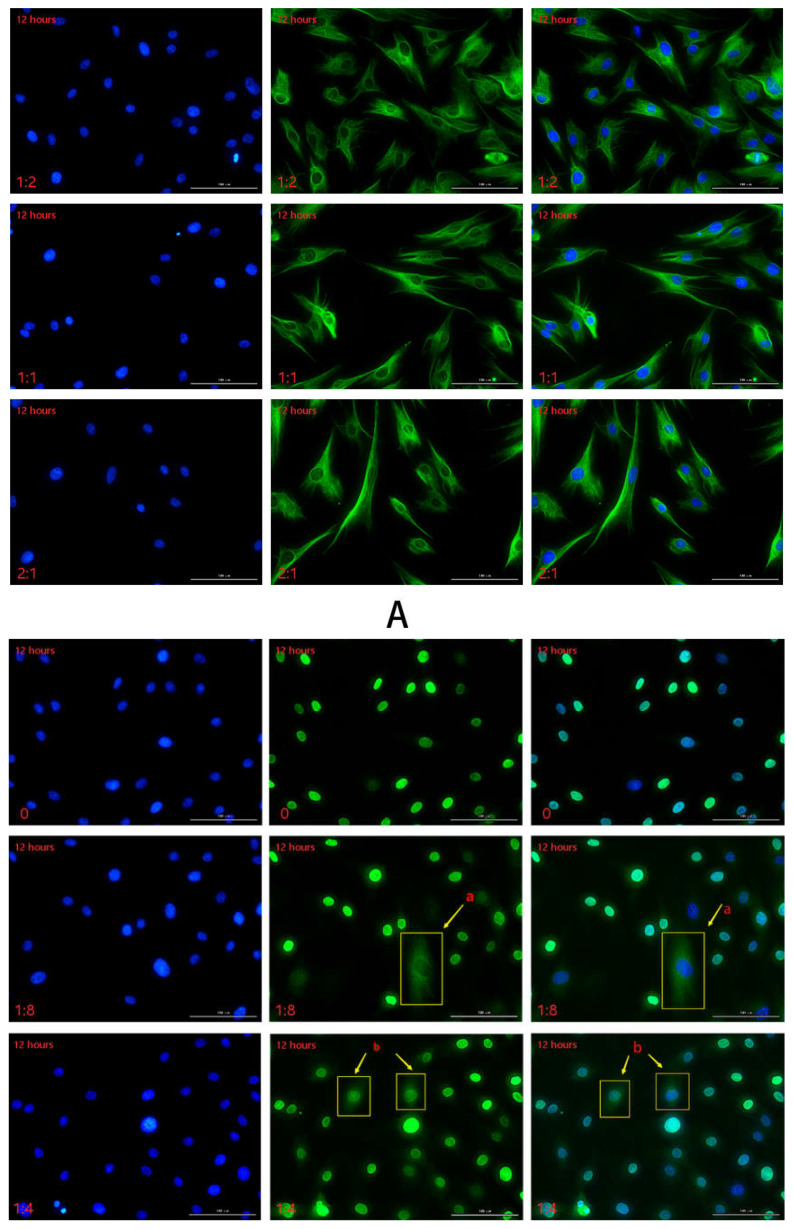

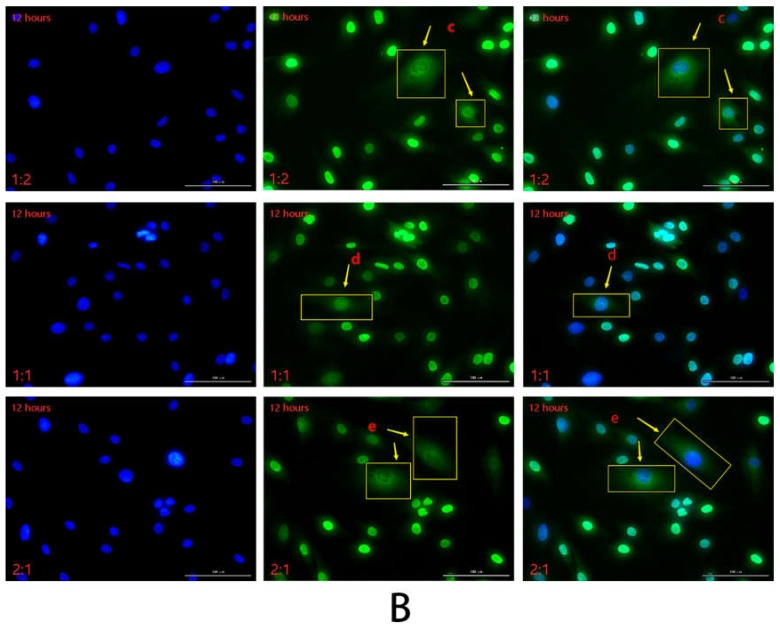
The immunofluorescence staining map of VEGFA and FGF2 proteins secreted by HSFs in the cell co-culture system. (**A**) The immunofluorescence staining of VEGFA protein secreted by HSFs after cell co-culture for 12 h. (**B**) The immunofluorescence staining of FGF2 protein secreted by HSFs after cell co-culture for 12 h. Scale bar: 100 μm.

**Figure 8:**
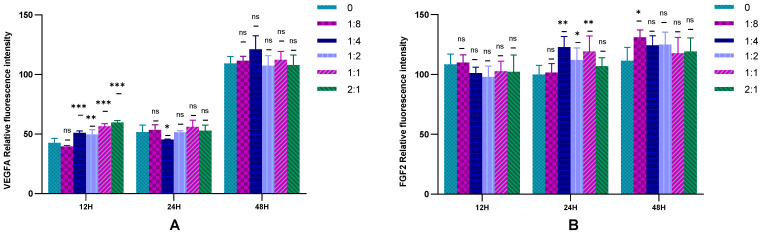
Immunofluorescence statistical map of HSFs in cell co-culture system. (**A**) VEGFA protein and (**B**) FGF2 protein. ns: *p* > 0.05, *: *p* < 0.05, **: *p* < 0.01, ***: *p* < 0.001.

The VEGFA and FGF2 secreted by HSFs in the cell co-culture system were detected by immunofluorescence, and the results are shown in Figure 7, Figure 8 and Figures S3–S6. Figure 7A is the result of immunofluorescence staining of VEGFA in HSFs. The green part is the VEGFA protein secreted by HSFs, and the blue is the nucleus of HSFs stained with DAPI. As can be seen from Figure 7A, VEGFA protein is mainly distributed in the cytoplasm of HSFs, enveloping the nuclei of HSFs. Figure 7B shows the immunofluorescence staining map of FGF2 protein secreted by HSFs. The green part is FGF2 protein, and the blue part is the nucleus. As can be seen from Figure 7B, when there are only HSFs in the cell co-culture system (HVECs:HSFs = 0), and the culture time is 12 h, FGF2 protein is only distributed in the nucleus, there is no distribution of FGF2 protein in the cytoplasm. However, with the addition of HVECs in the cell co-culture system, the FGF2 protein secreted by HSFs was transferred from the nucleus to the cytoplasm (such as Figure 7B(a–e), Figures S5(f–k) and S6(i–o)), and this phenomenon intensified with the increase of the ratio of HVECs and the prolongation of cell co-culture time. This phenomenon indicates that HVECs have an effect on the intracellular distribution of FGF2 protein secreted by HSFs in the cell co-culture system. The fluorescence intensities of the proteins in Figure 7 and Figures S4–S6 are counted, and the results are shown in Figure 8. As can be seen from Figure 8, when the cell co-culture time is 12 h, HVECs can promote HSFs to secrete VEGFA protein. When the cell co-culture time is 24 h, HVECs can promote HSFs to secrete FGF2 protein. The results show that HVECs could promote the secretion of VEGFA and FGF2 proteins in HSFs when it was co-cultured with HVECs.

#### 2.3.3. The Secretion of HSFs Proteins VEGFA and FGF2 Detected by WB

**Figure 9:**
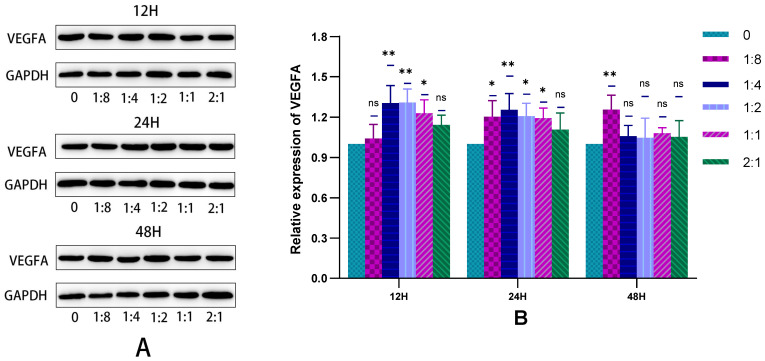
In the cell co-culture system, the WB detection map of VEGFA protein secreted by HSFs. (**A)** The WB map of VEGFA protein secreted by HSFs after cell co-culture for 12 h, 24 h, and 48 h. (**B**) The statistical results of relative expression of VEGFA protein secreted by HSFs. ns: *p* > 0.05, *: *p* < 0.05, **: *p* < 0.01.

**Figure 10:**
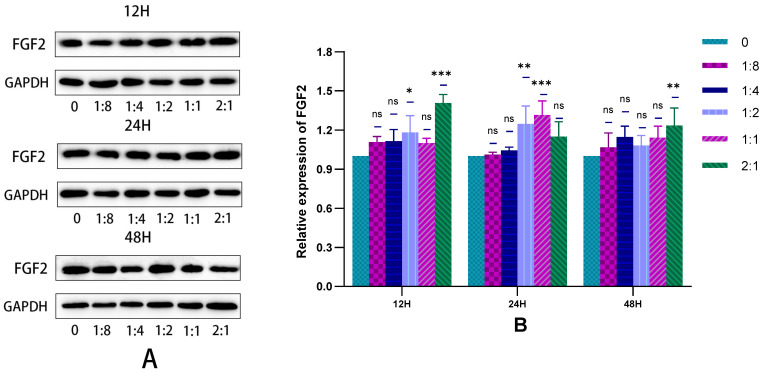
In the cell co-culture system, the WB detection map of FGF2 protein secreted by HSFs. (**A**) The WB map of FGF2 protein secreted by HSFs after cell co-culture for 12 h, 24 h, and 48 h. (**B**) The statistical results of relative expression of FGF2 protein secreted by HSFs. ns: *p* > 0.05, *: *p* < 0.05, **: *p* < 0.01, ***: *p* < 0.001.

In this experiment, the WB technique was used to detect the VEGFA and FGF2 proteins secreted by HSFs in the cell co-culture system, and the results are shown in Figure 9 and Figure 10. As can be seen from Figure 9B, when cells were co-cultured for 12 h to 24 h, HVECs significantly promote the secretion of VEGFA protein by HSFs. This promoting effect was most obvious when the ratio of HVECs to HSFs was 1:4. As can be seen from Figure 10B, when the cell co-culture time is 12 h and 24 h, HVECs can significantly promote HSFs to differentiate into FGF2 protein, and this promoting effect will increase with the increase of HVECs ratio in the cell co-culture system. The results showed that HVECs could promote the secretion of VEGFA and FGF2 proteins in HSFs during co-culture.

### 2.4. Results of HSFs Growth on Tissue Engineering Scaffolds

**Figure 11:**
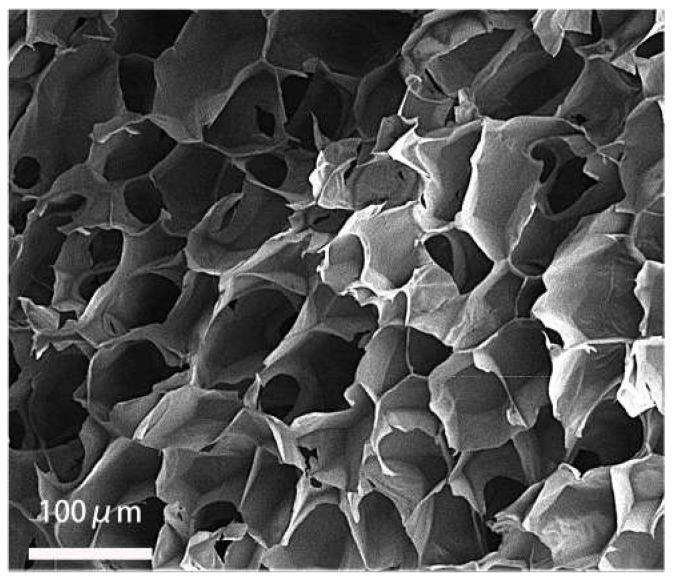
The scanning electron microscopic image of chitosan demethylated sodium cellulose cell scaffolds.

**Figure 12:**
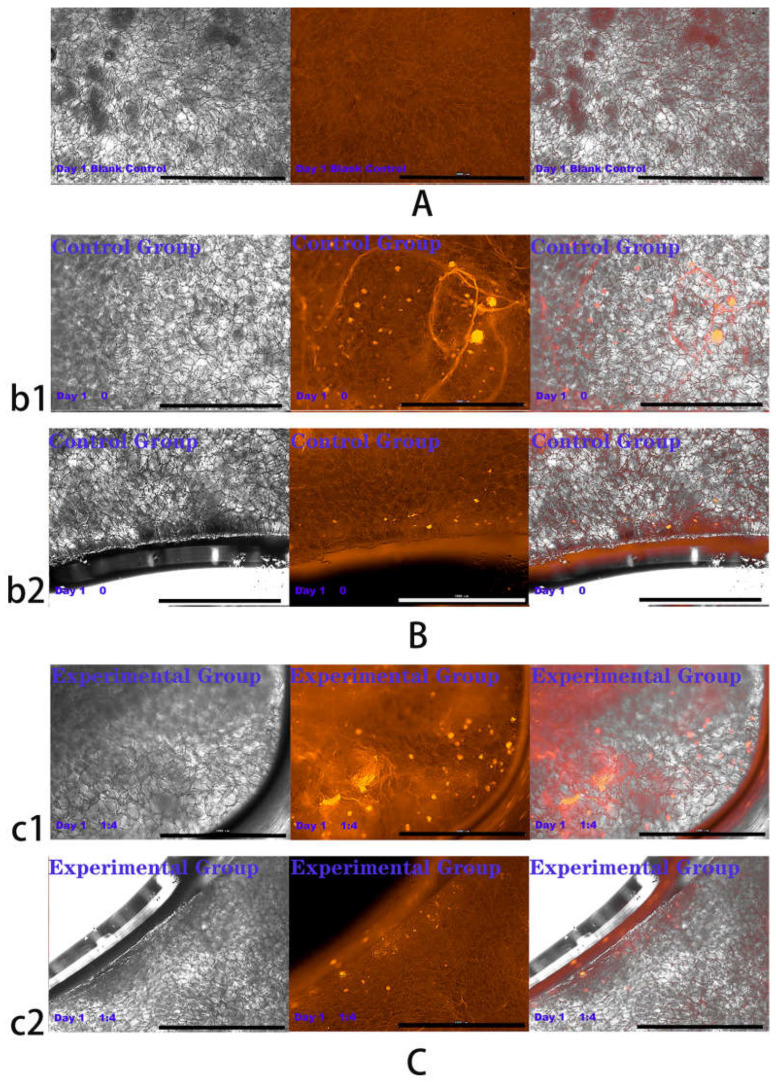
Cells were co-cultured for 1 day. Growth of HSFs labeled with amino graphene quantum dots on tissue engineering scaffolds. b1 and c1 are fluorescence maps of HSFs growing on the surface of tissue engineering scaffolds. b2 and c2 are fluorescence maps of HSFs growing on the cross section of the tissue engineering scaffolds. (**A**) Blank group, (**B**) control group, (**C**) experimental group. From left to right are the bright field diagram, the fluorescent field diagram, and the sum diagram of the bright field and the fluorescent field. Scale bar: 1000 μm.

**Figure 13:**
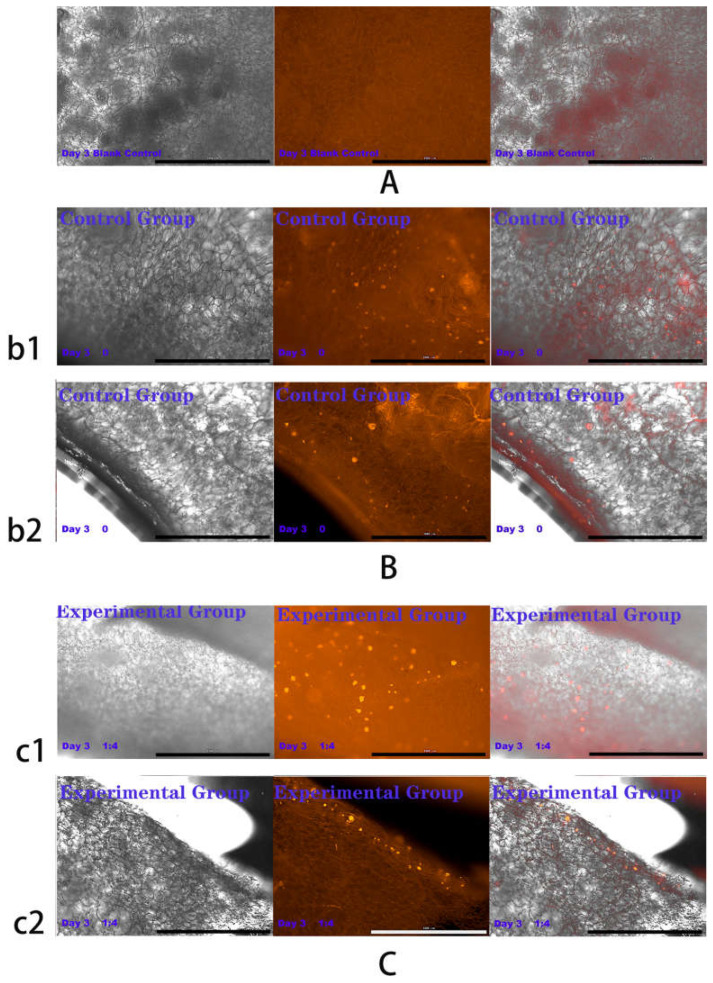
Cells were co-cultured for 3 days. Growth of HSFs labeled with amino graphene quantum dots on tissue engineering scaffolds. b1 and c1 and are fluorescence maps of HSFs growing on the surface of tissue engineering scaffolds. b2 and c2 are fluorescence maps of HSFs growing on the cross section of the tissue engineering scaffolds. (**A**) Blank group, (**B**) control group, (**C**) is experimental group. From left to right are the bright field diagram, the fluorescent field diagram, and the sum diagram of the bright field and the fluorescent field. Scale bar: 1000 μm.

**Figure 14:**
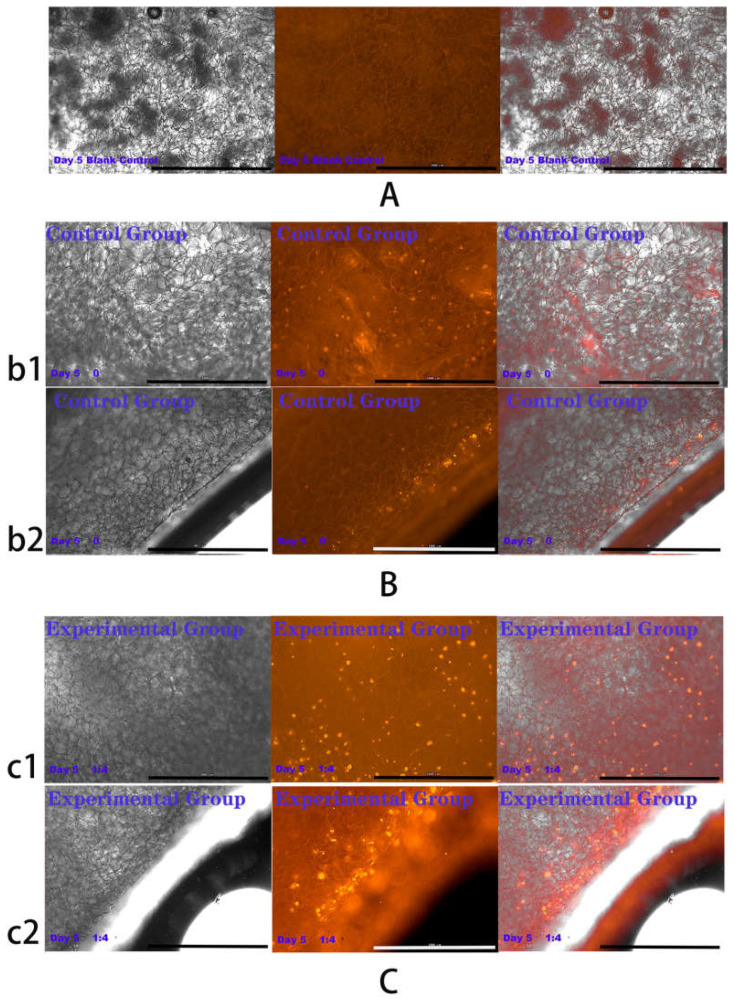
Cells were co-cultured for 5 days. Growth of HSFs labeled with amino graphene quantum dots on tissue engineering scaffolds. b1 and c1 and are fluorescence maps of HSFs growing on the surface of tissue engineering scaffolds. b2 and c2 are fluorescence maps of HSFs growing on the cross section of the tissue engineering scaffolds. (**A**) Blank group, (**B**) control group, (**C**) is experimental group. From left to right are the bright field diagram, the fluorescent field diagram, and the sum diagram of the bright field and the fluorescent field. Scale bar: 1000 μm.

**Figure 15:**
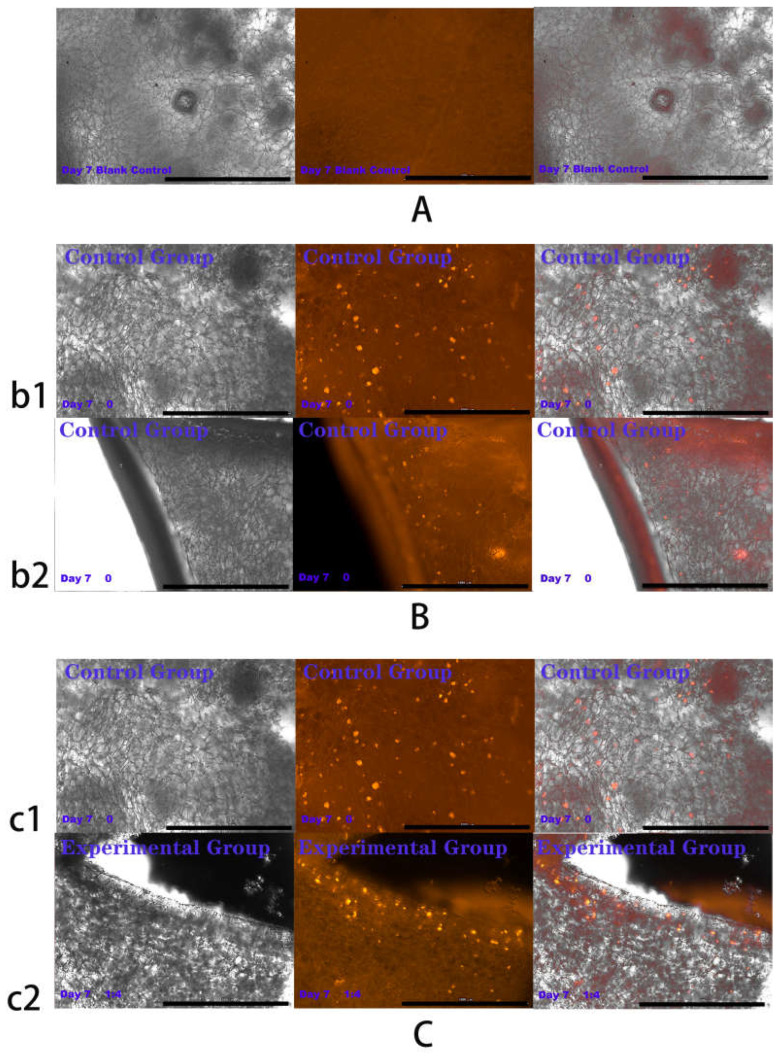
Cells were co-cultured for 7 days. Growth of HSFs labeled with amino graphene quantum dots on tissue engineering scaffolds. b1 and c1 and are fluorescence maps of HSFs growing on the surface of tissue engineering scaffolds. b2 and c2 are fluorescence maps of HSFs growing on the cross section of the tissue engineering scaffolds. (**A**) Blank group, (**B**) control group, (**C**) is experimental group. From left to right are the bright field diagram, the fluorescent field diagram, and the sum diagram of the bright field and the fluorescent field. Scale bar: 1000 μm.

**Figure 16:**
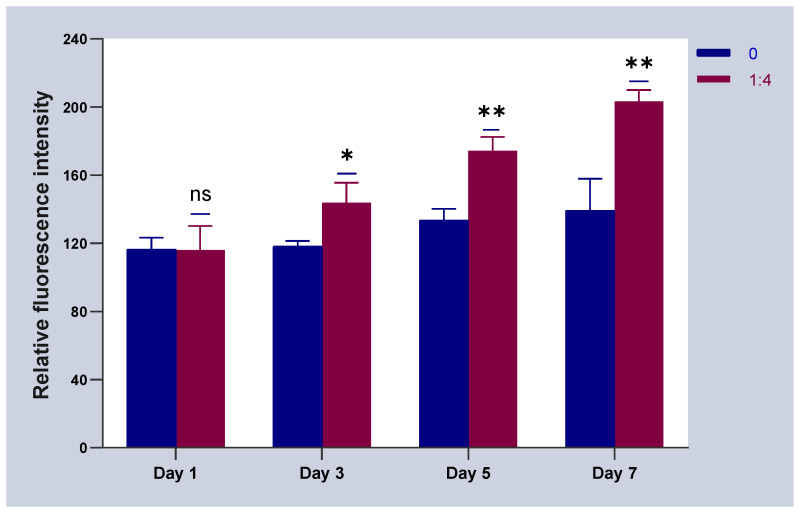
The statistical chart of fluorescence intensity of HSFs grown on the tissue engineering scaffolds. ns: *p* > 0.05, *: *p* < 0.05, **: *p* < 0.01.

The above experiments showed that in co-culture, HVECs could promote the migration of HSFs and the secretion of vascularization factors (VEGFA and FGF2) in different degrees. Based on the experimental data, the co-culture ratio of HVECs:HSFs at 1:4 was selected and applied to the tissue engineering scaffolds. Tissue engineering scaffolds was chitosan demethylcellulose sodium scaffolds prepared by references [11]. Its scanning electron microscope (SEM) results are shown in the Figure 11. The results of the SEM showed that the three-dimensional scaffolds had a honeycomb structure, which is similar to the geometric structure of the three-dimensional cell scaffold prepared after the decellularization of human skin. Such a honeycomb geometry has the conditions for HSFs to grow on it [12]. The HSFs (labeling with amino graphene quantum dots in advance, the marking method is obtained from my previously published paper [13]) and HVECs were inoculated at 1:4 (HVECs:HSFs) on tissue engineering scaffolds as the experimental group. The group without inoculation of HSFs and HVECs was used as the blank control group. The control group was only inoculated with the same number of HSFs as the experimental group but not HVECs. After being cultured for a period of time, the distribution of HSFs on the tissue engineering scaffold was detected by means of the fluorescence properties of HSFs on the 1st, 3rd, 5th, and 7th days respectively. The results are shown in Figure 12, Figure 13, Figure 14, Figure 15 and Figure 16. The orange-red dots in the Figure 12, Figure 13, Figure 14 and Figure 15 are HSFs labeled with amino graphene quantum dots. From the Figure 12, Figure 13, Figure 14 and Figure 15, with the increase of cell co-culture time, the distribution range of HSFs on the surface and cross section of tissue engineering scaffolds in the experimental group was larger than that in the control group. The fluorescence intensity of the HSFs grown on the tissue engineering scaffolds was counted, and the results are shown in Figure 16. As can be seen from Figure 16, on the 3rd day, the fluorescence intensity of the experimental group inoculated with HVECs in the cell co-culture system was higher than that of the control group without HVECs. On the 5th and 7th days, the fluorescence intensity of the experimental component fiber cells was significantly higher than that of the control group. The results of the application of HVECs and HSFs to the engineering scaffolds showed that the proliferation of HSFs could be promoted on the tissue engineering scaffolds after co-culture with HVECs. The result of preliminary application in engineering scaffolds showed the advantages of co-culture to tissue engineering.

## 3. Discussion

As the first protective barrier of the human body, the skin plays a role in resisting external invasion [14,15]. Wound healing after skin injury is a complex physiological process, including inflammatory reaction, tissue regeneration, functional reconstruction, and so on [16,17,18]. In the process of skin repair, fibroblasts secrete related factors and synthesized collagen, the abnormal secretion of these factors may lead to the formation of scar tissue [19]. Therefore, the related biological behavior of fibroblasts plays an important role in the repair of injured skin [20]. In the process of skin repair, whether a good capillary system can be formed is also the key to skin repair [21]. Vascular endothelial growth factor (VEGF) is one of the most effective angiogenic factors in the skin [22,23], including VEGFA, VEGFB, and so on. Angiogenesis is a complex step, including endothelial cell proliferation, migration, extracellular matrix degradation, and the coordination of a variety of growth factors [24], while VEGF plays a key role in the whole process, which can promote endothelial cell mitosis, and induce proliferation and migration. Improving the continuous utilization rate of vascular endothelial growth factor is an important factor in the construction of vascularized tissue-engineered skin [25]. The FGF2 secreted by fibroblasts is also one of the main vascularization factors [26,27], which not only plays an important role in the formation and repair of blood vessels, but also accelerates the growth of fibroblasts and endothelial cells in skin wounds, and promotes granulation tissue formation, epithelialization, and tissue remodeling [28]. A large area of skin defect makes it difficult for the body to repair itself [29]. The emergence of tissue-engineered skin provides a new way to solve this clinical problem [30]. It is expanded and cultured on biomaterials with a very small number of seed cells in vitro, and then transplanted into the body to treat large areas of defective skin tissue [31,32]. In recent years, the tissue engineering construction of single cells has been unable to meet the requirements of tissue structure simulation, tissue physiological function reconstruction, tissue stability maintenance, and so on [33]. Especially in the process of clinical treatment, the difficulty of vascularization of tissue engineering has become a difficult problem in the treatment of skin tissue engineering [34,35]. Based on the above facts, researchers tend to use multicellular culture to construct seed cell systems [36]. In the aspect of cell co-culture, scholars have become aware of the advantages of co-culture cell systems in recent years. PASCHOS [37] proposed that in the co-culture system, many types of cells can control the behavior between cells through the signal transduction of soluble factors. The study of the interaction between cells in the co-culture system can provide a theoretical reference for the application of the co-culture model to tissue engineering and to solve the problem of skin defect treatment. In this paper, HSFs, the main cells of the human skin dermis, were selected as co-cultured seed cells. In order to promote the proliferation, migration, and secretion of vascularization factors of HSFs, HVECs were introduced as companion cells in cell co-culture systems. The Transwell chamber was used as the carrier of non-contact co-culture model to study the effect of endothelial cells on the biological behavior of fibroblasts in a co-culture environment.

The above studies showed that through the establishment of co-culture system, the HVECs could effectively promote the migration of HSFs and up-regulate the migration factor HMGB1. The HVECs could also promote the secretion and expression of VEGFA and FGF2 in HSFs. Combined with the experimental results, the co-culture model with the ratio of HVECS:HSFs = 1:4 was introduced into the three-dimensional tissue engineering scaffolds. The experimental results showed that the proliferation rate of HSFs on the three-dimensional tissue engineering scaffold was faster than that of the control group with only HSFs. These experimental results provided a good theoretical basis for the introduction of the co-culture model into tissue engineering. In the follow-up study, it was also found that HVECs could significantly promote the secretion of I and IV collagen of HSFs. The I and IV collagen is the collagen that makes up the dermis of human skin. It is closely related to wound repair and wound healing [38]. It is suggested that the co-culture of HVECs and HSFs can promote the construction of HSFs in skin tissue. The deficiency of this experiment lies in the application. In this paper, the effect of HVECs on the biological behavior of HSFs in co-culture was deeply studied, and then the results of co-culture were simply applied to three-dimensional scaffolds, the proliferation of HSFs on the cell scaffolds was detected under the condition of co-culture. Further experiments can detect the secretion of vascularization factor and collagen of HSFs on the cell scaffolds during co-culture to identify the advantages of HSFs co-cultured with HVECs in tissue engineering scaffolds. At the same time, animal experiments can also be designed to implant the cell scaffolds after co-culturing into the animals to detect the tissue formation in the animals to further explain the experimental results.

## 4. Materials and Methods

### 4.1. Reagents and Apparatus

The HSFs and HVECs were donated by the Shanxi Bethune Hospital, Dulbecco’s modified Eagle medium (DMEM) high-sugar medium was purchased from Cytiva (Marlborough, MA, USA), fetal bovine serum (FBS) was purchased from Gibco (Waltham, MA, USA), CCK-8 kit was purchased from Prell (Shanghai, China), total RNA extraction kit was purchased from Promega (Beijing, China), PrimeScript™ RT with gDNA Eraser kit and TB Green Premix Ex Taq II kit was purchased from Takara (Kusatsu, Japan), mouse anti-human GAPDH antibody (AG019) was purchased from Beyotime (Haimen, China). Rabbit anti-human VEGFA antibody (AB214424), rabbit anti-human FGF-2 antibody (AB92337), rabbit anti-human HMGB1 antibody (AB18256), sheep anti-rabbit HRP antibody (AB6721), sheep anti-mouse HRP antibody (AB6789), recombinant rabbit anti-human anti-VEGFA antibody (AB52917), recombinant rabbit anti-human anti-FGF2 antibody (AB208687), recombinant rabbit anti-human anti-HMGB1 antibody (AB79823), and sheep anti-rabbit IgG H&L antibody (AB150077) were purchased from Abcam (Cambridge, UK). PCR primers were synthesized by Shenggong Biochem (Shanghai, China).

Bio Tek Living cell workstations (Winooski, VT, USA); Beckman Coulter cryogenic high-speed refrigerated centrifuge (Brea, CA, USA); electrophoretic trough, transmembrane trough, and electrophoretic apparatus (Beijing, China); StepOnePlus™ real-time PCR system (ThermoFisher, Waltham, MA, USA); Tanon 6400 automatic chemiluminescence image analysis system (Shanghai, China); Thermo Forma CO2 incubator (Massachusetts, USA). Olympus IX70 Fluorescence Inverted Microscope (Tokyo, Japan), JET Biofil Transwell (24-hole plate, membrane pore diameter 8 μm and 6-hole plate, membrane pore diameter 8 μm) were used in the study.

### 4.2. Establishment of Cell Co-Culture Model

**Figure 17:**
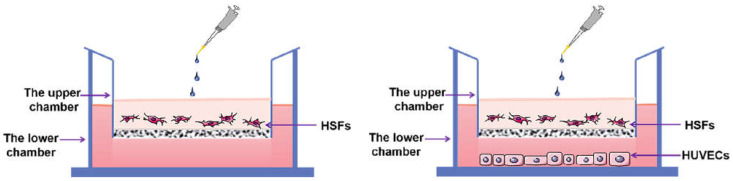
The HSFs-HVECs cell co-culture model. The picture on the left shows the control group with the proportion of HVECs being 0.

The cell co-culture model was established by using a Transwell migration chamber with a six-well plate and a membrane pore diameter of 3 μm (Figure 17). The 2 × 10^5^ HSFs were inoculated in the upper chamber of Transwell and the HVECs were inoculated in the lower chamber. The ratio of HVECs to HSFs was 0, 1:8, 1:4, 1:2, 1:1, and 2:1. The cell co-culture models of these six proportions were divided into one group, and the culture time of each group was 12 h, 24 h, and 48 h, respectively. During the culture period of each group, the culture medium was not changed. The following experiments were carried out on the HSFs in the above co-culture model: CCK8 cell activity test; Transwell cell migration ability test; RT-qPCR, immunofluorescence, WB to detect migration factor HMGB1 and vascularization factors VEGFA, FGF2.

### 4.3. Detection of HSFs Activity

In the experiment, CCK8 was used to detect the activity of HSFs in the above 4.2 Transwell cell co-culture system, and the Transwell upper chamber was removed and placed in a new 6-well plate. The culture medium containing 10% CCK8 was added and placed in the incubator for 2 h, the upper culture medium was sucked out, and the amount of 100 ul per empty was added to the 96-well plate. The absorbance of the culture medium was measured at a wavelength of 450 nm using a microplate reader.

### 4.4. Experiment on the Migration Ability of HSFs

#### 4.4.1. Transwell Cell Migration Experiment

The HSFs were inoculated into the upper chamber of the Transwell chamber (24-well plate, 8.0 μm aperture). The HVECs suspensions of 0, 1:8, 1:4, 1:2, 1:1, and 2:1 were added into the hole of the lower chamber. The 24-well Transwell chamber was cultured in a cell incubator. After 12 h, the upper chamber of Transwell was taken out, fixed with paraformaldehyde, stained with 1% crystal violet dye, and cleaned with PBS. The HSFs that had not migrated in the upper chamber of Transwell were gently wiped off with a cotton swab. The HSFs migrated to the inferior intima of Transwell chamber were observed by a light microscope. According to the same experimental method, the migration of HSFs was detected when the co-culture time was 24 h and 48 h.

#### 4.4.2. Detection of Migration Factor HMGB1 Gene Expression by RT-qPCR

The total RNA of HSFs was extracted by total RNA extraction kit and reverse transcribed to obtain cDNA. Real-time fluorescence quantitative PCR was used to detect the effect of HVECs on the expression of the HMGB1 gene in HSFs with different proportions and culture times. The relative amount of gene expression (GAPDH expression as the relative quantitative standard) was calculated by 2^−∆∆ct^. The primers used in the gene were synthesized by Shanghai Shenggong, and the related sequence is shown in Table 1.

#### 4.4.3. The Expression of Migration Factor HMGB1 Protein was Detected by WB and Immunofluorescence

The HSFs in Transwell chamber in 4.2 were lysed with protein lysate to prepare total protein samples. The protein concentration was determined by the BCA method, electrophoresed on 10% SDS-PAGE gel, and transferred to the PVDF membrane by electric transfer method. 5% nonfat milk powder was blocked at room temperature, then primary antibodies GAPDH (dilution ratio 1:2000) and HMGB1 (AB18256) (dilution ratio 1:2000) were added and incubated on a shaker at 4 ℃ overnight. The primary antibodies were removed, washed with TBST, and the secondary antibodies were added and incubated at room temperature for 2 h. The secondary antibodies used were 1:10,000 dilution of HRP labeled sheep anti-mouse antibody and sheep anti-rabbit antibody. ECL chemiluminescence detection was carried out using Image J software for gray analysis and calculation. Protein lysate configuration: 10 µL protease inhibitor cocktail, 10 µL phosphorylase inhibitor A solution, 10 µL phosphorylase inhibitor B solution, and 10 µL protease inhibitor lysates into 1 mL lysis buffer.

The HMGB1 protein secreted by HSFs in the co-culture system was detected by immunofluorescence. The HSFs were seeded in the lower chamber of a 6-well plate (3.0 μm pore size), and HVECs were seeded in the upper chamber of Transwell at the ratios of 0, 1:8, 1:4, 1:2, 1:1, and 2:1, and cultured for 12 h, 24 h, and 48 h. The HSFs at the bottom of the well plate was stained with immunofluorescence. The primary antibody (recombinant rabbit anti-human anti-HMGB1 antibody (AB79823)) was diluted at a ratio of 1:200, and the secondary antibody (sheep anti-rabbit IgGH&L antibody (AB150077)) was diluted at a ratio of 1:1000. The cell nucleus were localized by DAPI staining. The HSFs after immunofluorescence were photographed. ImageJ software was used for gray analysis and calculation.

### 4.5. Detection of HSFs Vascularization Factor-VEGFA and FGF2

#### 4.5.1. Detection of VEGFA and FGF2 Expression by RT-qPCR

The cDNA obtained from 4.3.2 was used to detect the expression of VEGFA and FGF2 in HSFs cultured with different proportion of HVECs for different times by RT-qPCR assay. The relative amount of gene expression (GAPDH expression as the relative quantitative standard) was calculated by 2^−∆∆CT^. The primers used in the gene were synthesized by Shanghai Shenggong, and the related sequence is shown in Table 2.

#### 4.5.2. Detection of VEGFA and FGF2 Expression by WB and Immunofluorescence

The total protein samples prepared in 4.3.3 were used to detect the protein expressions of HSFs vascularization factors-VEGFA and FGF2 by WB and immunofluorescence, respectively. The primary antibodies used for WB were GAPDH (AG019), VEGFA (AB214424), and FGF2 (AB92337), and the dilution ratio was 1:2000. The secondary antibodies were sheep anti-rabbit HRP antibody (AB6721) and sheep anti-mouse HRP antibody (AB6789), the dilution ratio was 1:10,000. The immunofluorescence primary antibodies were recombinant rabbit anti-human anti-VEGFA antibody (AB52917) and recombinant rabbit anti-human anti-FGF2 antibody (AB208687) with a dilution ratio of 1:200. The secondary antibody was sheep anti-rabbit IgG H&L antibody (AB150077) with a dilution ratio of 1:1000. Image J software was used for gray analysis and calculation of the detection results.

### 4.6. Detection of HSFs Growth on Tissue Engineering Scaffolds

**Figure 18:**
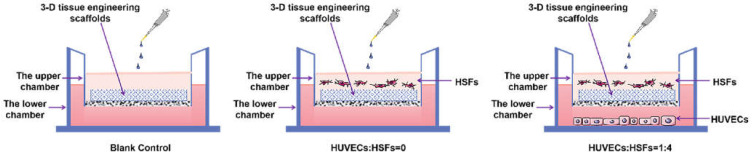
The Transwell model of cell co-culture.

According to the experiments from 4.3 to 4.5, the cell co-culture ratio was determined to be 1:4 to study the growth of HSFs on tissue engineering scaffolds (the tissue engineering scaffolds were the chitosan demethylcellulose sodium cell scaffolds prepared by our group). The specific experimental contents are as follows: the tissue engineering scaffolds are placed in the Transwell room, and the HSFs are inoculated on the tissue engineering scaffolds (HSFs are labeled with amino graphene quantum dots in advance), HVECs (HVECs:HSFs = 1:4) were inoculated into the lower chamber of Transwell, which was used as the experimental group. The HVECs were not inoculated into the lower ventricle in the control group, and other conditions were the same as those in the experimental group. In the blank control group, HVECs and HSFs were not inoculated, and other conditions were the same as those in the experimental group. The models of all experiments are shown in Figure 18. The growth of HSFs on tissue engineering scaffolds was detected by the fluorescence properties of HSFs at 1, 3, 5, and 7 days.

### 4.7. Statistical Analysis

Image J software and SPSS19.0 were used for image processing and data analysis, and GraphPad Prism8 software was used for drawing. All the experiments were repeated three times independently, and the data were expressed as mean ± standard deviation, single factor analysis of variance was used, LSD test was used for post-comparison. The difference was statistically significant (*p* < 0.05).

## Figures and Tables

**Table 1:** Primer sequences.

Gene Name	Forward Primer (F) Reverse Primer (R)
GAPDH	F: CAAGGGCATCCTGGGCTACACT R: CTCTCTCTTCCTCTTGTGCTCTTGC
HMGB1	F: ATGCTTCAGTCAACTTCTCAGA R: CATTTCTCTTTCATAACGGGCC

**Table 2:** Primer sequences.

Gene Name	Forward Primer (F) Reverse Primer (R)
GAPDH	F: CAAGGGCATCCTGGGCTACACT R: CTCTCTCTTCCTCTTGTGCTCTTGC
VEGFA	F: ATCGAGTACATCTTCAAGCCAT R: GTGAGGTTTGATCCGCATAATC
FGF-2	F: CATCAAGCTACAACTTCAA-GCA R: CCGTAACACATTTAGAAGCCAG

## Data Availability

Not applicable.

## Supplementary Materials

The following supporting information can be downloaded at: https://www.mdpi.com/article/10.3390/ijms232213995/s1, Figure S1: The HMGB1 immunofluorescence staining map secreted by HSFs after co-culture for 12 h; Figure S2: The HMGB1 immunofluorescence staining map secreted by HSFs after co-culture for 48 h; Figure S3: The VEGFA immunofluorescence staining map secreted by HSFs after co-culture for 24 h; Figure S4: The VEGFA immunofluorescence staining map secreted by HSFs after co-culture for 48 h; Figure S5: The FGF2 immunofluorescence staining map secreted by HSFs after co-culture for 12 h; Figure S6: The FGF2 immunofluorescence staining map secreted by HSFs after co-culture for 48 h.
