# Supplementary material for: Human Vascular Endothelial Cells Promote the Secretion of Vascularization Factors and Migration of Human Skin Fibroblasts under Co-Culture and Its Preliminary Application
Source: Int J Mol Sci. 2022 Nov 13;23(22):13995. doi: 10.3390/ijms232213995 (PMC9697737; doi:10.3390/ijms232213995)
Supplement: Supplementary file 1 [file ijms-23-13995-s001.zip › ijms-2013444-supplementary.pdf]

Supplementary Materials:

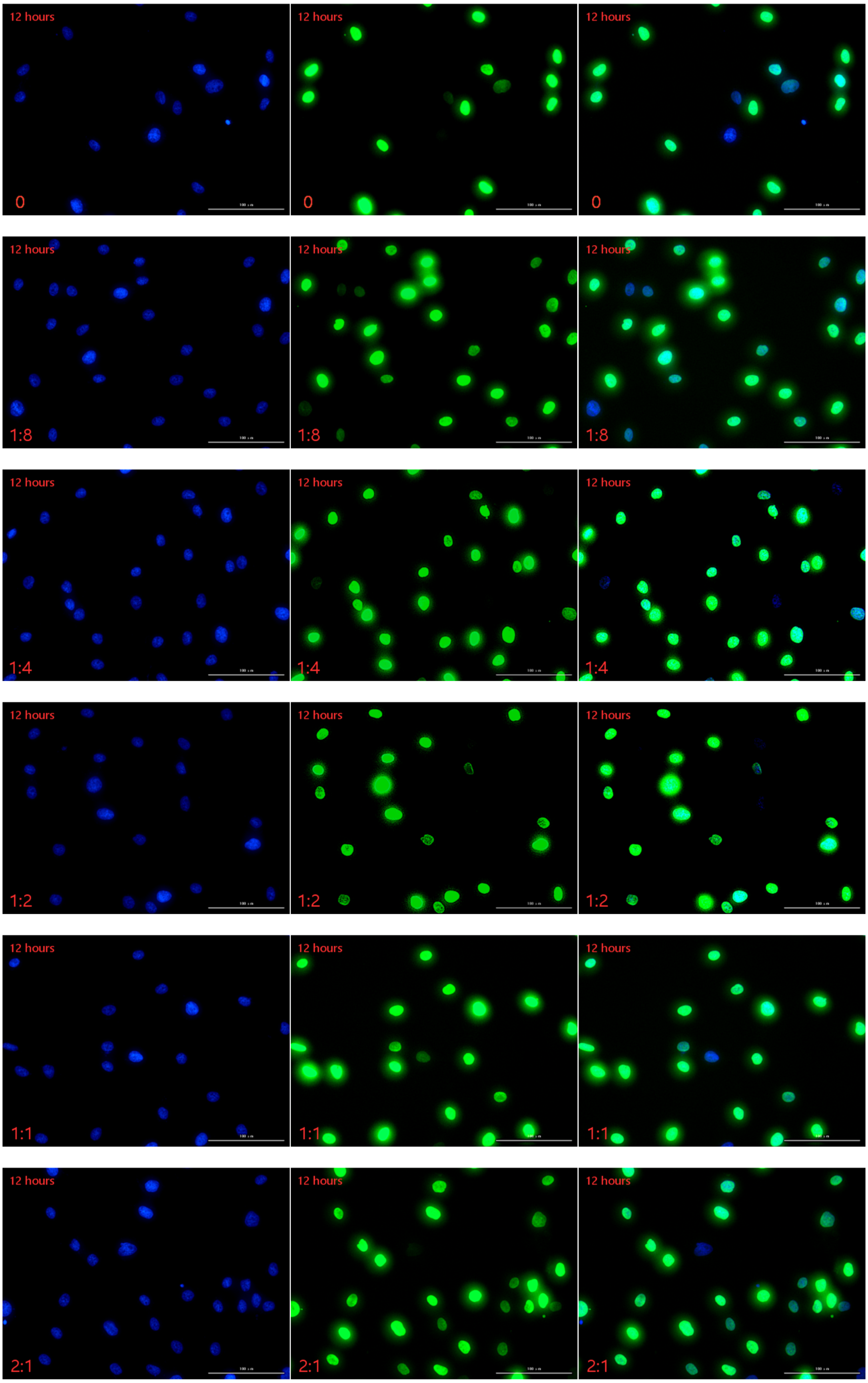

Figure S1: The HMGB1 immunofluorescence staining map secreted by HSFs after co-culture for 12 h.

In the cell co-culture system, HSFs were stained with immunofluorescence.The figure shows the immunofluorescence staining of HMGB1 protein secreted by HSFs after cell co-culture for 12 hours. Scale bar :100 μm.

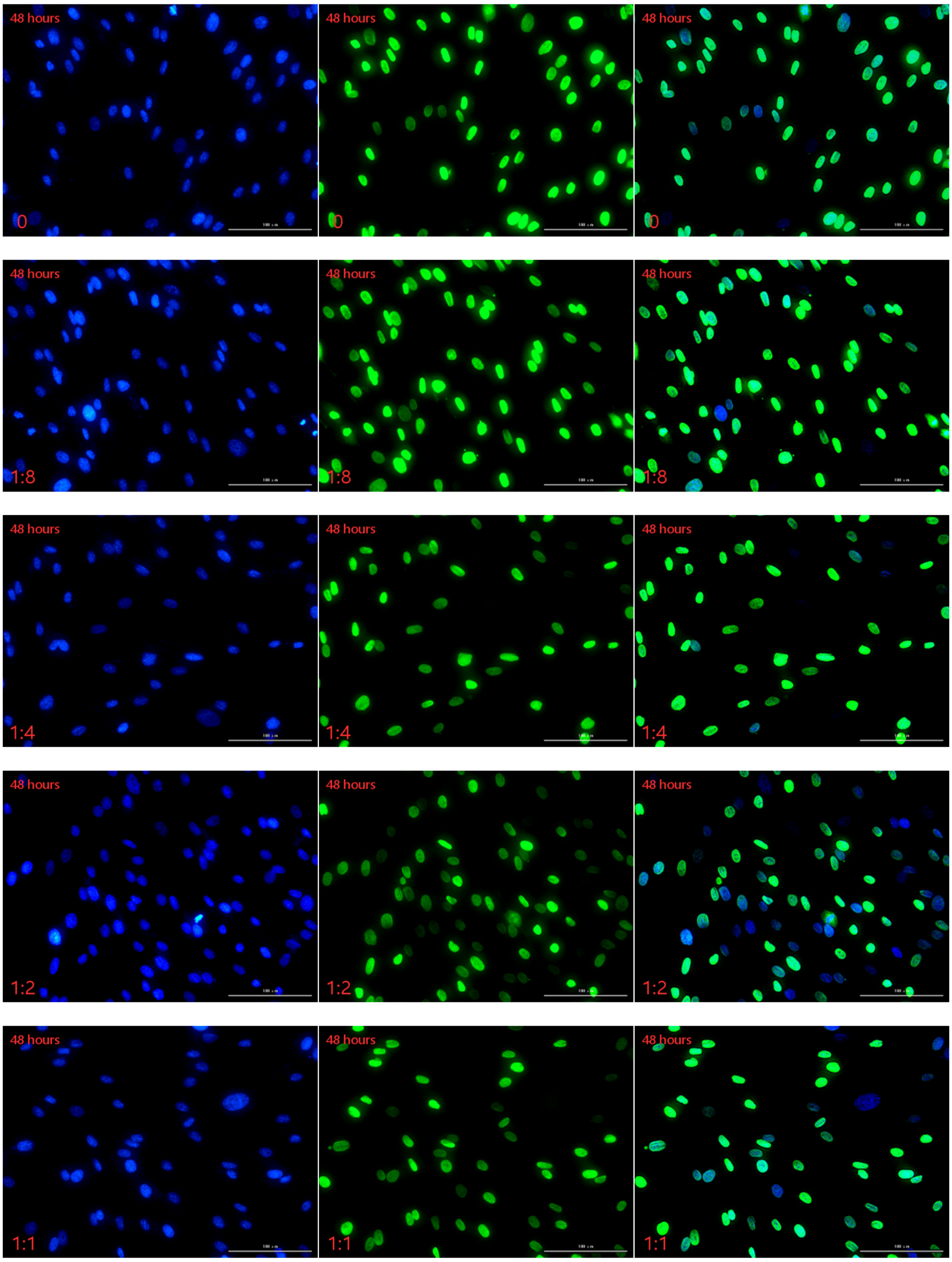

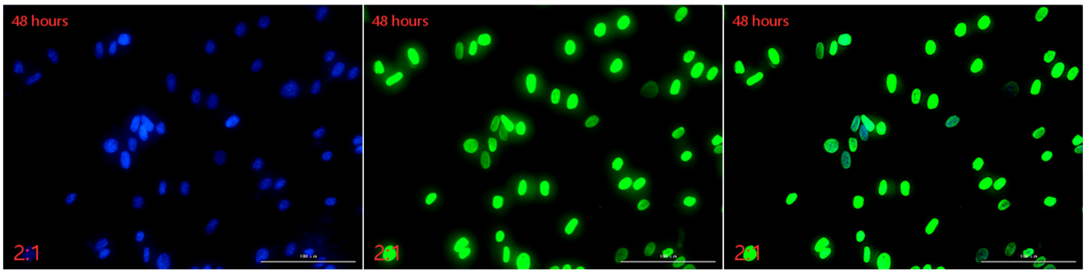

Figure S2: The HMGB1 immunofluorescence staining map secreted by HSFs after co-culture for 48 h.

In the cell co-culture system, HSFs were stained with immunofluorescence.The figures shows the immunofluorescence staining of HMGB1 protein secreted by HSFs after cell co-culture for 48 hours. Scale bar :100 μm.

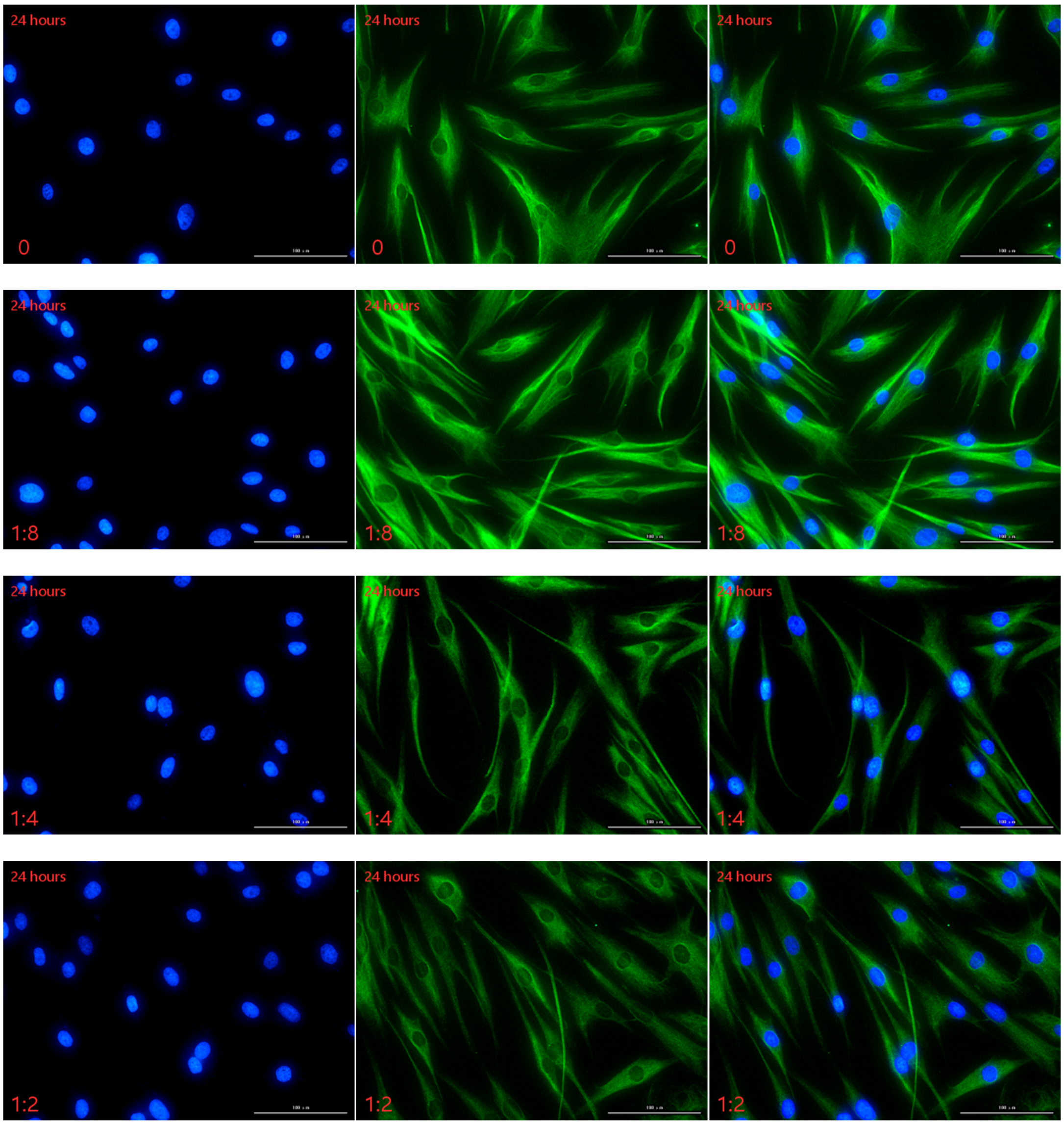

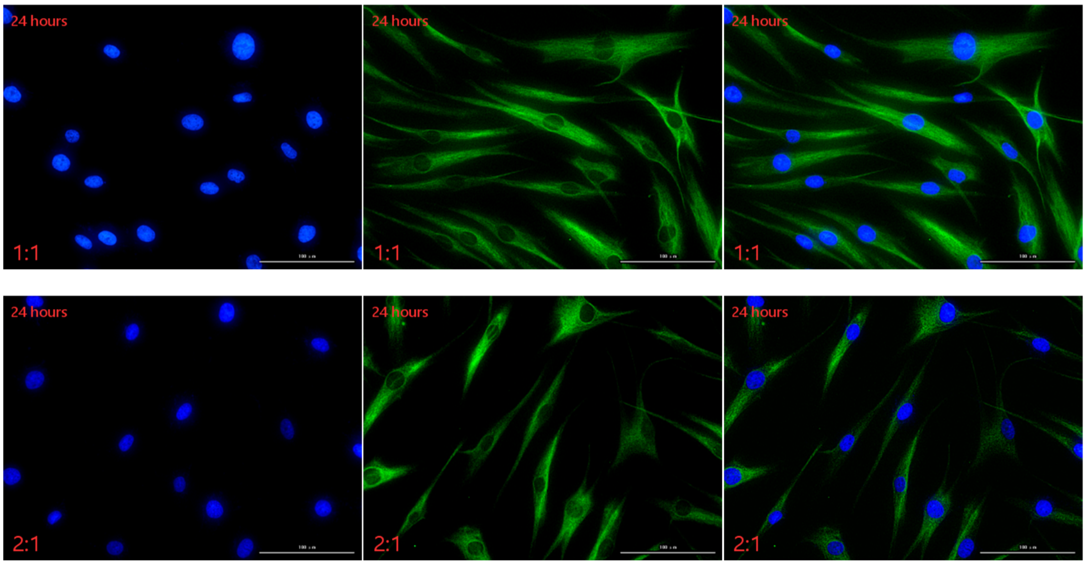

Figure S3: The VEGFA immunofluorescence staining map secreted by HSFs after co-culture for 24 h.

The immunofluorescence staining map of VEGFA secreted by HSFs in the cell co-culture system. The figures shows the immunofluorescence staining of VEGFA protein secreted by HSFs after cell co-culture for 12h. Scale bar: 100 μm.

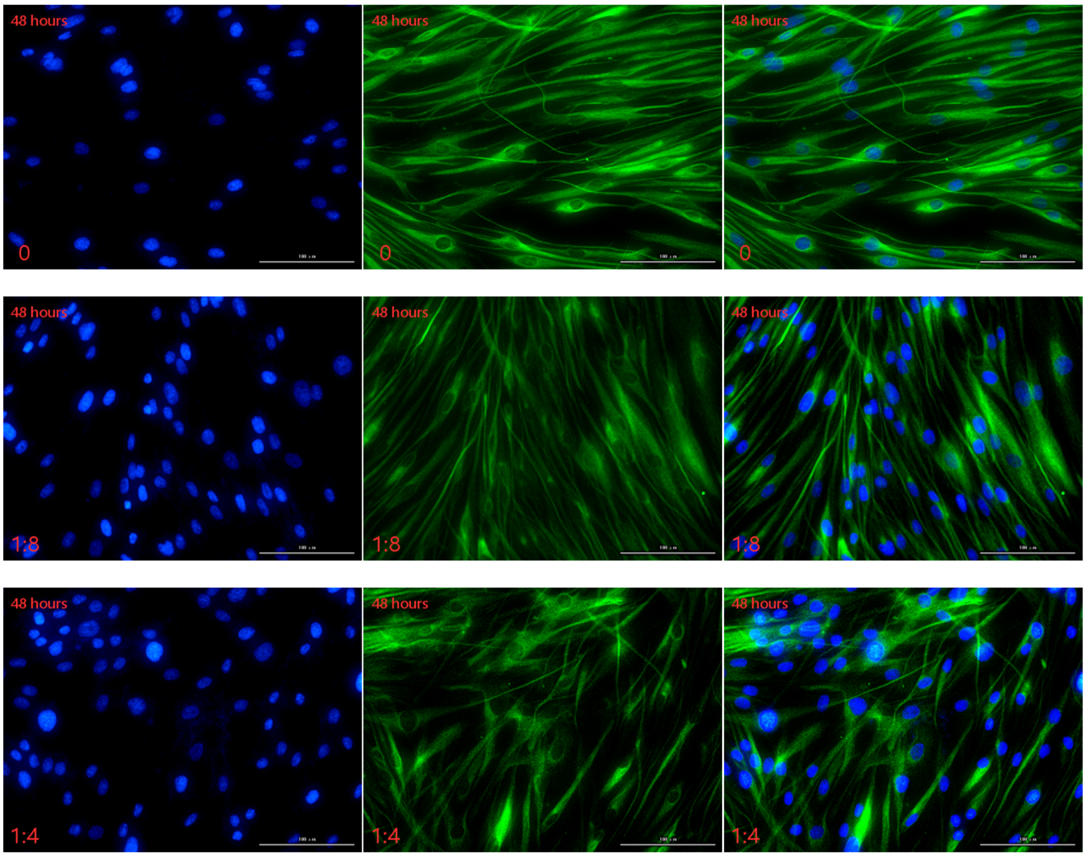

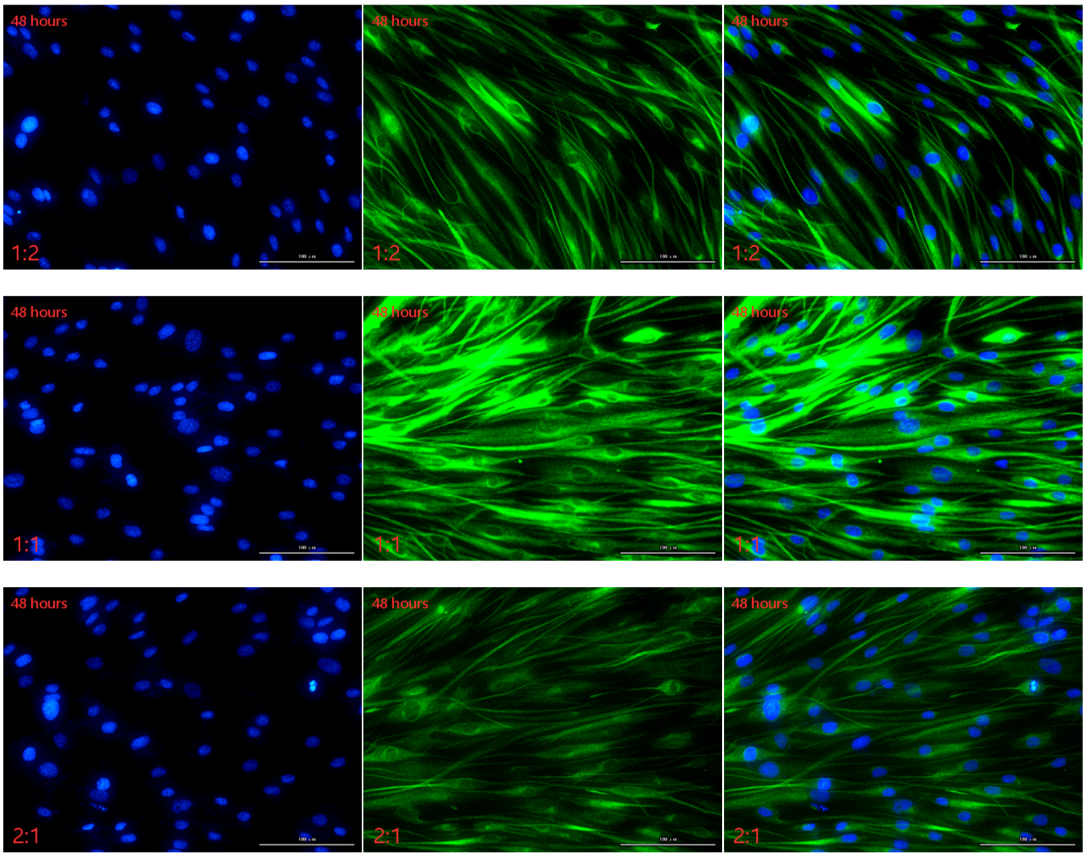

Figure S4: The VEGFA immunofluorescence staining map secreted by HSFs after co-culture for 48 h.

The immunofluorescence staining map of VEGFA secreted by HSFs in the cell co-culture system. The figures shows the immunofluorescence staining of VEGFA protein secreted by HSFs after cell co-culture for 48 h. Scale bar: 100 μm.

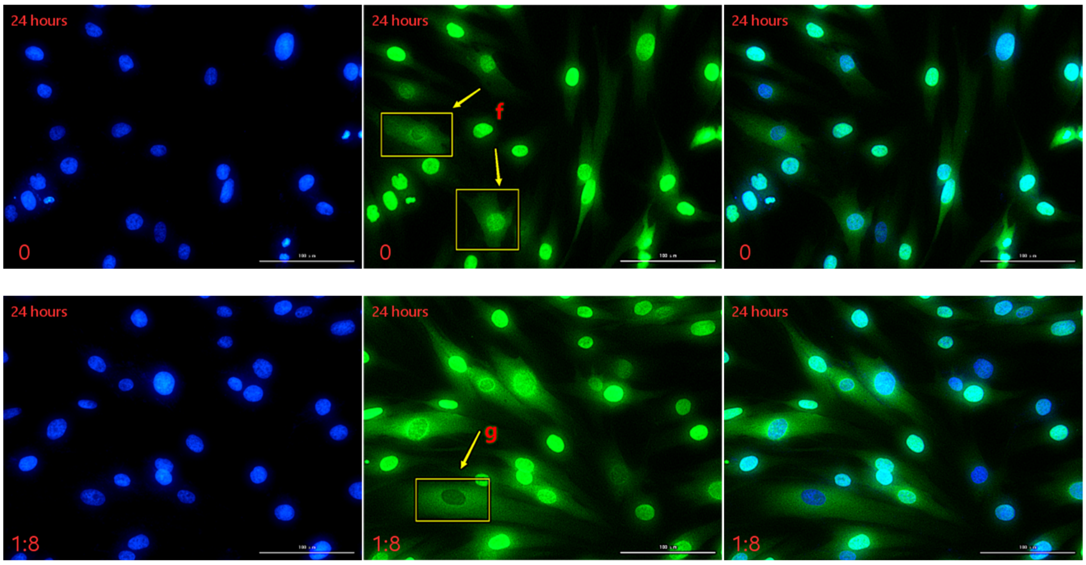

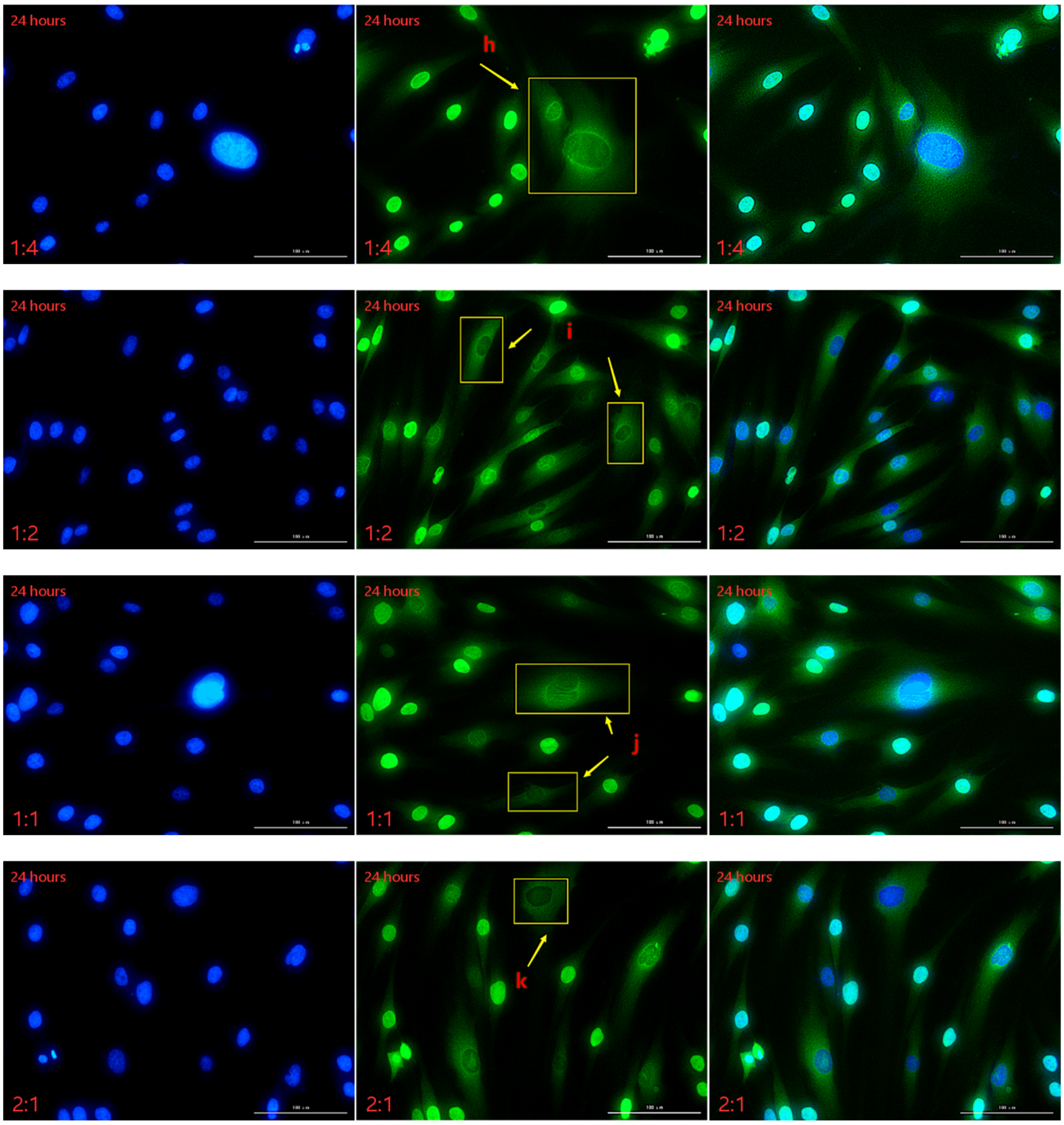

Figure S5: The FGF2 immunofluorescence staining map secreted by HSFs after co-culture for 12 h.

The immunofluorescence staining map of FGF2 secreted by HSFs in the cell co-culture system. The figures shows the immunofluorescence staining of FGF2 protein secreted by HSFs after cell co-culture for 12h. Scale bar: 100 μm.

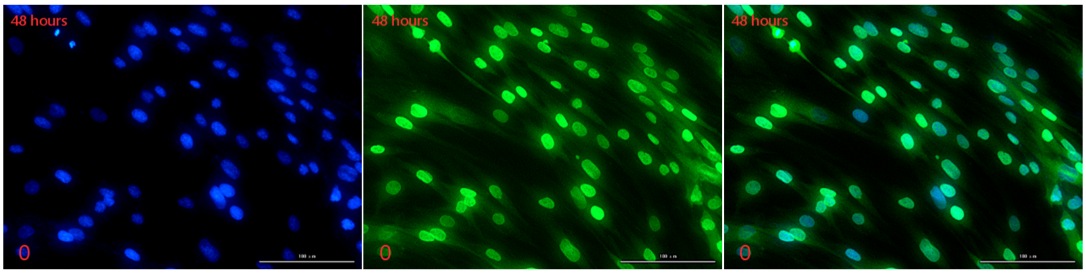

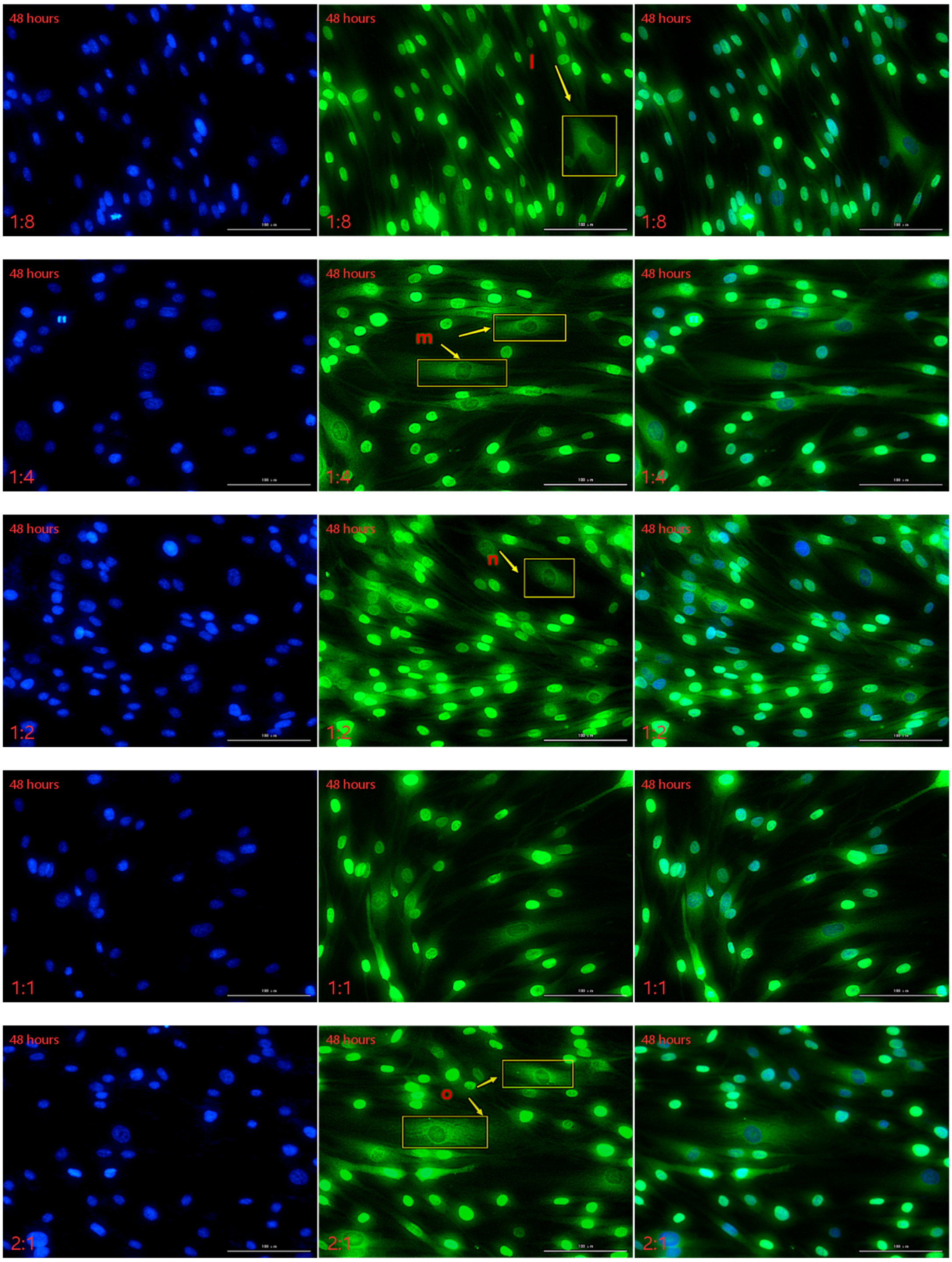

Figure S6: The FGF2 immunofluorescence staining map secreted by HSFs after co-culture for 48 h.

The immunofluorescence staining map of FGF2 secreted by HSFs in the cell co-culture system. The figures the immunofluorescence staining of FGF2 protein secreted by HSFs after cell co-culture for 48 h. Scale bar: 100 μm.

Gel electrophoresis original pictures:

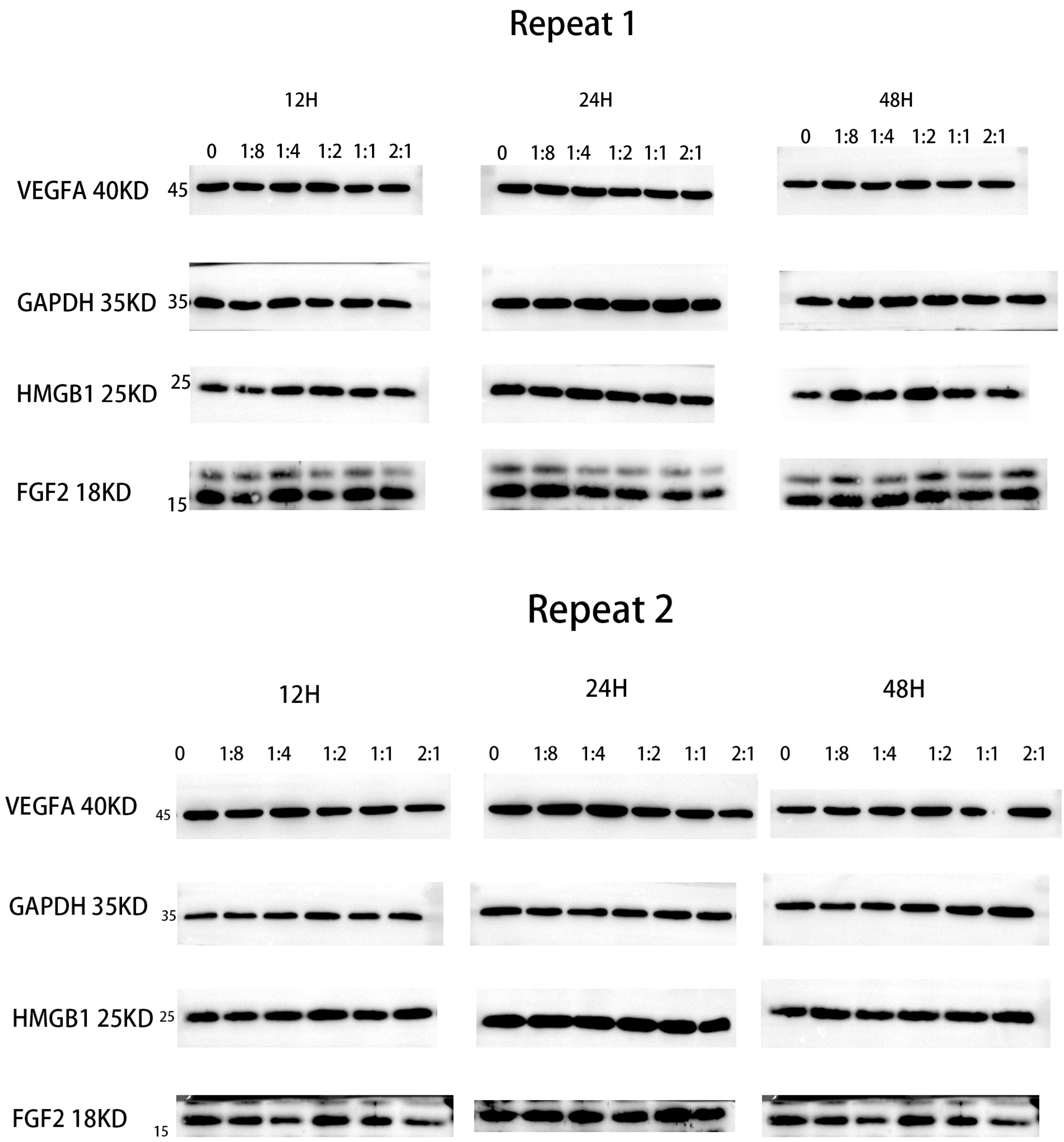

Repeat 3

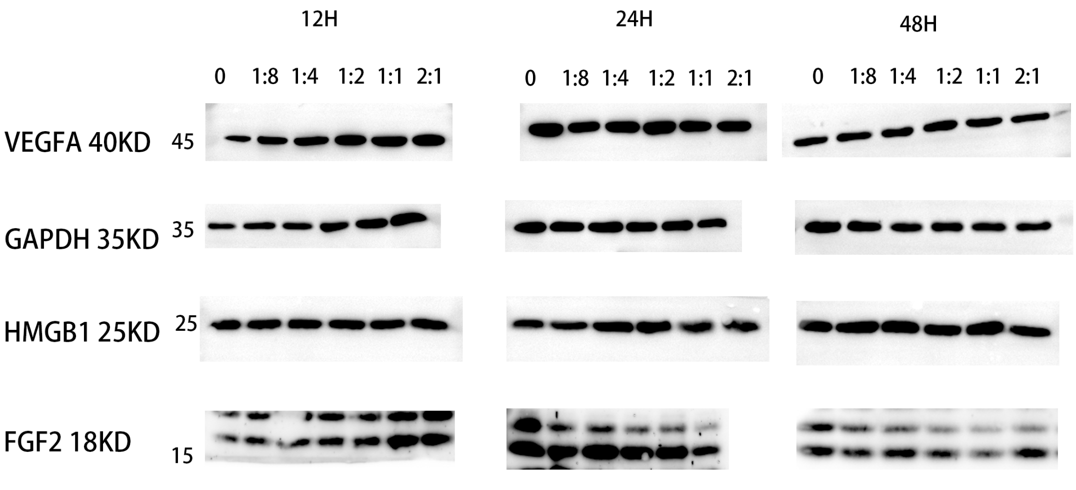
12H
24H
48H
0
1:8
1:4
1:2
1:1
2:1
VEGFA 40KD
45
GAPDH 35KD
35
HMGB1 25KD
25
FGF2 18KD
15
